# Limited effects of macro-nutrient ratios on thiamin content and transfer in phytoplankton and copepods

**DOI:** 10.1093/plankt/fbad004

**Published:** 2023-02-01

**Authors:** Emil Fridolfsson, Sanna Majaneva, Samuel Hylander

**Affiliations:** Department of Biology and Environmental Sciences, Centre for Ecology and Evolution in Microbial model Systems – EEMiS, Linnaeus University, Kalmar SE-39182, Sweden; Department of Biology and Environmental Sciences, Centre for Ecology and Evolution in Microbial model Systems – EEMiS, Linnaeus University, Kalmar SE-39182, Sweden; Norwegian University of Science and Technology, Department of Biology / Trondhjem Biological Station, Trondheim NO-7491, Norway; Department of Biology and Environmental Sciences, Centre for Ecology and Evolution in Microbial model Systems – EEMiS, Linnaeus University, Kalmar SE-39182, Sweden

**Keywords:** B-vitamins, micronutrient, trace element, primary producer, zooplankton

## Abstract

Vitamin B_1_ (thiamin) is primarily produced by bacteria, phytoplankton and fungi in aquatic food webs and transferred to higher trophic levels by ingestion. However, much remains unknown regarding the dynamics this water-soluble, essential micronutrient; e.g. how it relates to macronutrients (carbon, nitrogen and phosphorous). Nutrient limitation has been found to be related to periods of thiamin deficiency as well as in models. Hence, thiamin transfer to copepods from three phytoplankton species from different taxa was investigated, along with the effect of various nutrient regimes on thiamin content. Nutrient levels did not affect thiamin content of phytoplankton nor the transfer to copepods. Instead, phytoplankton displayed species-specific thiamin and macronutrient contents and whilst a higher thiamin content in the prey lead to higher levels in copepods, the transfer was lower for *Skeletonema* compared to *Dunaliella* and *Rhodomonas*. In all, thiamin transfer to copepods is not only dependent on thiamin content of the prey, but also the edibility and/or digestibility is of importance. Thiamin is essential for all organisms, and this study offers insights into the limited effect of macronutrients on the dynamics and transfer of thiamin in the aquatic food webs.

## INTRODUCTION

Phytoplankton, as part of the base of marine food webs, provide energy and nutrients for primary consumers such as herbivorous zooplankton, and therefore also to higher trophic levels, in the form of organic compounds. Beside the quantitative effects of resource availability, growth, and development of herbivores, like copepods, can be influenced by phytoplankton species composition (Nejstgaard *et al.*, 2001) and the relative amount of essential nutrients in their food, i.e. phytoplankton elemental ratios (Sterner and Elser, 2002). Variability in phytoplankton nutrient requirements and their acquisition mechanisms play a central role in defining phytoplankton community structure (Tilman *et al.*, 1982).

The stoichiometric homeostasis, structural N:P ratio, has been shown to be species-specific, and thus changes in supply rates of nutrients and their concentrations will lead to modifications in the community structure (Rhee and Gotham, 1980). Photosynthetic organisms appear to be quite flexible in their growth rate and chemical composition exhibiting “weak stoichiometric homeostasis,” and can adjust their ratios depending on availability of nutritional elements such as nitrogen (N) or phosphorus (P) in the environment. On the other hand, zooplankton tend to be more nutrient-rich compared to phytoplankton and generally exhibit strong stoichiometric homeostasis, meaning that they maintain a given elemental composition even when consuming food with a broad variety of C:N:P ratios. This often-observed plasticity in phytoplankton stoichiometry can lead to elemental mismatches between consumers’ demand for essential nutrients and the relative availability of their prey (Sterner and Elser, 2002; Malzahn *et al.*, 2007; Boersma *et al.*, 2008; Persson *et al.*, 2010). Species are known to possess various physiological mechanisms to help compensate for stoichiometric imbalance. However, as with most traits, there are tradeoffs or costs for these physiological compensatory mechanisms. A large stoichiometric imbalance between the primary producer and consumer generally results in decreased growth efficiency (Sterner and Elser, 2002; Mitra and Flynn, 2005; Hessen *et al.*, 2013), reproduction (Jónasdóttir, 1994), and could alter the consumer community composition (Karpowicz *et al.*, 2019). Similarly, the imbalance may reflect underlying allocations to important molecules (e.g. vitamins and lipids), which are closely associated with key traits such as growth rate and reproduction. Stoichiometric imbalances between adjacent trophic levels in food webs could have implications on the rates and efficiencies with which energy and elements are processed in ecosystems. In aquatic environments, thiamin (vitamin B_1_) is mainly produced by bacteria, phytoplankton and fungi and is transferred in the food web through ingestion by organisms to higher trophic levels (Webb *et al.*, 2007; Combs, 2012; Sañudo-Wilhelmy *et al.*, 2014). As all B-vitamins, thiamin is water-soluble which implies that storage is limited and organisms that do not produce thiamin *de novo*, known as thiamin auxotrophs, require a constant supply of thiamin (Jurgenson *et al.*, 2009).

The Baltic Sea food web is, like many other systems, highly productive; blooms of phytoplankton have increased as well as the primary production (Bonsdorff *et al.*, 2002). However, despite the surplus of food many organisms at higher trophic levels, both fish and bird species, display symptoms of nutritional deficiency and lack of important molecules (Bengtsson *et al.*, 1999; Balk *et al.*, 2009; Balk *et al.*, 2016; Engelhardt *et al.*, 2020). One of the most well-documented thiamin deficiency syndromes in aquatic systems is the M74-syndrome in the Baltic Sea salmon, where as many as 70–80% of the females in some years produce offspring with the syndrome leading to high offspring mortality during the yolk-sac fry stage (Bengtsson *et al.*, 1999; Mikkonen *et al.*, 2011; Keinänen *et al.*, 2012; Börjesson, 2016; ICES, 2020). In addition to the M74 syndrome in the Baltic Sea, thiamin deficiency have been reported for salmonines from the Great Laurentian Lakes (early mortality syndrome, EMS) (Marcquenski and Brown, 1997) and the New York Finger Lakes (Cayuga syndrome) (Fisher *et al.*, 1995). As thiamin deficiency can result in several sublethal effects in addition to direct mortality, the term thiamin deficiency complex (TDC) was proposed by Riley and Evans (2008). The peak M74 incidences have been shown to occur after the Baltic Sea underwent phases with lower salinity with relatively low phosphate and silicate concentrations and high availability of nitrogen, and these bottom-up forces affected the complex connectivity among the trophic levels (Majaneva *et al.*, 2020). Furthermore, a modeling study showed that bottom-up effects e.g. nutrient availability, can constrain the transfer of thiamin from producers to consumers in aquatic food webs (Ejsmond *et al.*, 2019). Since nutrient requirements, N:P ratio, within phytoplankton are species-specific it can be hypothesized that some species show reduction of food quality also in a form of lower thiamin content when growing in a nutrient limited environment (Ejsmond *et al.*, 2019; Majaneva *et al.*, 2020). This change in the food quality, will then affect the higher trophic levels as the thiamin uptake of grazing copepods will be reduced in nutrient limited systems.

The ability to produce thiamin (thiamin prototrophy) is not evenly distributed among bacteria and phytoplankton, and the auxotrophy level in different phytoplankton phyla varies greatly. On average, thiamin auxotrophy level among phytoplankton is ∼25%, where some phyla display thiamin auxotrophy of 86%, while some phyla are 100% thiamin prototrophic (Carlucci and Bowes, 1970; Croft *et al.*, 2006; Tang *et al.*, 2010).Thiamin content is highly variable among phytoplankton (Brown *et al.*, 1999; Sylvander *et al.*, 2013; Gutowska *et al.*, 2017; Fridolfsson *et al.*, 2018, 2020). Filamentous cyanobacteria, like *Dolichospermum* sp., *Aphanizomenon flos-aquae* and *Nodularia spumigena* have previously been reported to have relatively higher thiamin content compared to other classes like Bacillariophyceae, Chlorophyceae, Dinophyceae, Prymnesiophyceae and Cryptophyceae, which had lower thiamin levels (Gutowska *et al.*, 2017; Fridolfsson *et al.*, 2018, 2020; Sylvander *et al.*, 2013). Interestingly, the high thiamin content of cyanobacteria does not automatically mean high transfer to copepods, shown in both feeding experiments and field conditions (Fridolfsson *et al.*, 2018, 2020; Fridolfsson *et al.*, 2019). Yet, the effect of nutrient alteration on vitamin content in phytoplankton and following uptake on zooplankton is not well known.

For the Baltic Sea area, bioassays have shown that primary production is mostly P-limited in the Bothnian Bay (Tamminen and Andersen, 2007; Andersson *et al.*, 1996) and mostly N-limited in the Kattegat (Graneli *et al.*, 1990), but nutrient limitation patterns switch during seasons (Tamminen and Andersen, 2007), in relation to proximity to freshwater sources (Pitkänen and Tamminen, 1995), and during blooms of cyanobacteria (Lignell *et al.*, 2003; Nausch *et al.*, 2004). Changes in N:P or C: nutrient ratios may alter phytoplankton species composition (Prins *et al.*, 2012) as well as the quality of phytoplankton as a food source for zooplankton. In the phytoplankton community in the Baltic Sea, anthropogenic stressors have been suggested to have led to a regime shift from a stage dominated by diatoms and a copepod species *Pseudocalanus acuspes*, to a stage dominated by dinoflagellates, cyanobacteria and other copepod species, like *Acartia* spp. (Möllmann *et al.*, 2008; Casini *et al.*, 2009). As well, it has been suggested that while diatoms with higher thiamin content are beneficial for overall thiamin production in the system compared to other phytoplankton groups (Van Nieuwerburgh *et al.*, 2004; Sylvander *et al.*, 2013; Fridolfsson *et al.*, 2020), this regime shift could be the cause of symptoms of deficiency syndromes displayed at the higher trophic levels. Beside the effects on total nutrient loads, human impact on biogeochemical cycles has strongly changed the stoichiometry of nutrient availabilities in coastal areas worldwide. Nevertheless, due to species-specific differences in the stoichiometric balances we hypothesize that there is variance in thiamin production between N- and P-limited phytoplankton species as well as between different phytoplankton groups. Hence, in this study, we conducted a set of experiments with phytoplankton cultivated in different nutrient conditions, and zooplankton fed with these phytoplankton to study how the nutrient imbalance effects the thiamin production and uptake.

## METHOD

Two separate experimental setups were used in spring 2016 to study the effect of nutrient stoichiometric imbalances in the water and phytoplankton and its potential effect on the thiamin uptake of copepods in Baltic Sea plankton communities. In Experiment I, we examined the effect of nutrient N:P supply ratios which were, respectively, lower or higher than Redfield ratios on the phytoplankton thiamin content and in Experiment II, we examined how the imbalance may reflect underlying allocations to thiamin concentration in the consumer.

### Phytoplankton cultures

Phytoplankton species included in the experiment were chosen on three premises: (1) species belonging to different taxonomic groups, (2) be limited both by N and P, (3) appropriate size to be consumed by *Acartia* spp., and (4) both thiamin auxotrophic (*Rhodomonas salina*) and prototrophic (*Skeletonema costatum* and *Dunaliella tertiolecta*). Non-axenic cultures of *D. tertiolecta* (Chlorophyceae, CCMP 1302, hereafter *Dunaliella*), *S. costatum* (Bacillariophyceae, KAC 44, hereafter *Skeletonema*) and *R. salina* (Cryptophyceae, KAC 30, hereafter *Rhodomonas*) from Kalmar Algae Collection (KAC), curated by Linnaeus University, were maintained in exponential growth phase as semi-continuous cultures on modified f/2 medium using filtered Baltic Sea water (0.2 μm membrane filters, 7 PSU). Macronutrients (nitrogen, phosphorous and silicate) and vitamins (cobalamin [0.4 nM], biotin [2 nM] and thiamin [296 nM]) were added according to Guillard (1975), while trace metals were added according to L1 media recipe (Guillard and Hargraves, 1993). Stock-cultures were grown under 100 μmol photons m^−2^ s^−1^ and a 16:8 h light: dark cycle, until inoculated to the experimental cultures with experimental growth media.

### Zooplankton collection

Prior to experiments, natural zooplankton communities were collected from the Linnaeus Microbial Observatory (LMO; N 56 55.85400, E 17 3.64200), described in Legrand *et al.* (2015) by oblique plankton net hauls from 30 m depth to the surface (50 cm, 200 μm mesh size). The zooplankton community mainly consisted of *Acartia* sp. with a few *Temora longicornis*, *Eurytemora* sp., and *Centropages hamatus* (Fridolfsson *et al.*, 2019). *Acartia* sp., being the most common copepod in the Baltic Sea system throughout the year (Fridolfsson *et al.*, 2019), was thus selected for the experiments. Samples were transported and stored in cooling boxes to the laboratory. Directly after sampling, *Acartia* sp. were sampled for thiamin, particulate organic carbon (POC) and particulate organic nitrogen (PON) as well as particulate organic phosphorus (POP). The first 24-36 h in the laboratory were used as an acclimatization period to the laboratory conditions (field temperature ~ 10°C, laboratory temperature 16°C). Prior to the experiment, adult *Acartia* sp. specimens were carefully picked and placed into 25-L tanks with filtered seawater (0.2 μm) with gentle aeration and fed with mixture of *Dunaliella, Skeletonema* and *Rhodomonas* at saturating levels every second day.

### Experimental setup

Two experiments were run consecutively in spring 2016, with one phytoplankton species at each time: *Dunaliella, Skeletonema* and *Rhodomonas*, respectively*.* In experiment I, three different nutrient regimes were used and was run in triplicates. The terms N-limited (Nlim) and P-limited (Plim) are used hereafter to indicate cultures that initially contained nutrient N:P supply ratios which were, respectively, lower or higher than Redfield ratios. To obtain nitrate or phosphate limitation, phytoplankton were grown in semi-continuous cultures with different nutrient conditions (N:P ratios); NP supplied with N and P in Redfield ratio medium at N:P = 16:1 (NO^3−^ = 58 μM; PO_4_^3−^ = 3.6 μM), Nlim with medium at N:P = 1.6:1 (NO^3−^ = 5.8 μM; PO_4_^3−^ = 3.6 μM) and Plim with medium at N:P = 160:1 (NO^3−^ = 58 μM, PO_4_^3−^ = 0.36 μM) according to the modifications and protocols of Berges *et al.* (2001) and Andersen (2005). Experiments were run for 7 days and samples for thiamin, POC, PON and POP were taken at the start and end.

In experiment II, a total of 135 *Acartia* sp. stage CV and females were kept in 1 L glass bottles with 0.2-μm filtered sea water. Copepods were fed phytoplankton from experiment I, and concentrations corresponded to bloom conditions during summer in the Baltic Sea (Legrand *et al.*, 2015). The 1 L bottles were randomly placed on a plankton wheel on a 16:8 light: dark cycle and 16°C with the speed 1 rpm. Experiments were run for 24 h, and samples for thiamin, POC, PON and POP were taken in the start and in the end of the experiment.

### Analysis

#### Phytoplankton abundance

Samples for phytoplankton abundance were preserved with 2% acidic Lugol’s solution, kept in the dark and counted using a Sedgwick-Rafter cell with an inverted microscope (Nikon TMS).

#### Analysis of thiamin

To measure thiamin levels, phytoplankton (10 mL) and 30 specimens of *Acartia* sp. (pooled) per replicate were transferred to Whatman GF/F filters. Filters were wrapped and immediately frozen and stored at −80°C until analyzed according to Pinto *et al.* (2002) with slight modifications as proposed by Sylvander *et al.* (2013) and Fridolfsson *et al.* (2018). Detection limit of the method is 15 fmol (Pinto *et al.*, 2002). Briefly, thawed samples were sonicated in 1–1.5 mL 0.1 M HCl with a Vibra-Cell sonicator (amplitude 40 for phytoplankton or 92 for copepod samples, respectively) on ice for 1.5 min with 1 s pulses. Extracts were centrifuged at 16 900$\times$g at 10°C during 10 min and 700 μL of the supernatant was centrifuged once more under the same conditions. Next, 600 μL of the supernatant was mixed with 550 μL MeOH, 300 μL 1 M NaOH and 50 μL freshly made 30 mM K_3_Fe(CN)_6_. Finally, the mix was filtered through a 0.45-μm PTFE/PP syringe filter. Standard solutions (1 μM) for the three types of thiamin, free thiamin (TF), thiamin monophosphate (TMP) and thiamin diphosphate (TDP) were prepared in 0.1 M HCl and aliquoted in a five-point standard series. Blank samples were prepared by mixing 600 μL 0.1 M HCl with the remaining chemicals. Standards and blanks were treated in the same way as samples, except for the sonication and centrifugation step. To ensure that the quantification was correct, a selection of samples was spiked with standards and the chromatograms inspected.

Thiamin samples were analyzed using a Hitachi Chromaster HPLC system with a Purospher®Star NH_2_ LiChroCART® column (5 μm particle size, 4.6 mm [I.D.]$\times$250 mm), protected by a Purospher®Star NH_2_ LiChroCART® guard column (5 μm particle size, 4 mm [I.D.]$\times$4 mm). Fluorescence was detected using an excitation wavelength of 375 nm and an emission wavelength of 450 nm. Injection volume was 100 μL with a flowrate of 1 mL min^−1^. Mobile phase consisted of MeOH and 0.1 M phosphate buffer (pH 7.4) at a ratio of 43:57 and the runtime was 24 min. Chromatograms were integrated using the software OpenLab (Agilent Technologies), and baselines were drawn automatically and inspected manually. Three types of thiamin were analyzed, TF, TMP and TDP and these values were summed up to get the total thiamin content (T_tot_).

#### Analysis of particulate carbon, nitrogen and phosphorous content

For POC/PON analysis, phytoplankton (10 mL) and 30 specimens of copepods (pooled) were transferred into pre-combusted Whatman GF/F glass fiber filters (475°C for 3 h), wrapped into H_2_O_2_ washed Eppendorf tubes and stored at −80°C until further analysis. Prior to analysis, filters were dried at 60°C for at least 24 h and stored in a desiccator. Carbon and nitrogen content was analyzed in a Perkin Elmer CHNS/O Analyzer 2400 Series II. POP was analyzed according to Solórzano and Sharp (1980) and SS-EN ISO 6878:2005 (2005). Due to methodological issues, POP analysis did not work for copepod samples.

### Data handling and statistical analyses

Statistical analyses were performed using R software version 3.4.2 (R Core Team, 2019). All graphs were created using the packages “ggplot2” (Wickham, 2009) and “gridExtra” (Auguie, 2017). Unless stated otherwise, the data are presented as average values and occasionally with standard deviation. Due to analytical problems, technical replicates for macro elemental composition are missing and accordingly, statistical investigation was not possible. Thiamin ratio was calculated by dividing the total thiamin content in the consumers (copepods) by the total thiamin content in the prey (phytoplankton), as previously described (Hairston and Hairston, 1993; Fridolfsson *et al.*, 2019; Fridolfsson *et al.*, 2020). A thiamin ratio > 1 implies that the thiamin content in copepods is higher than in its phytoplankton prey, indicating that copepods can assimilate and store thiamin, for some time at least. Notably, thiamin ratio does not consider retention efficiency or cellular demand of thiamin.

## RESULTS

### Thiamin content in phytoplankton and copepods

**Fig. 1:**
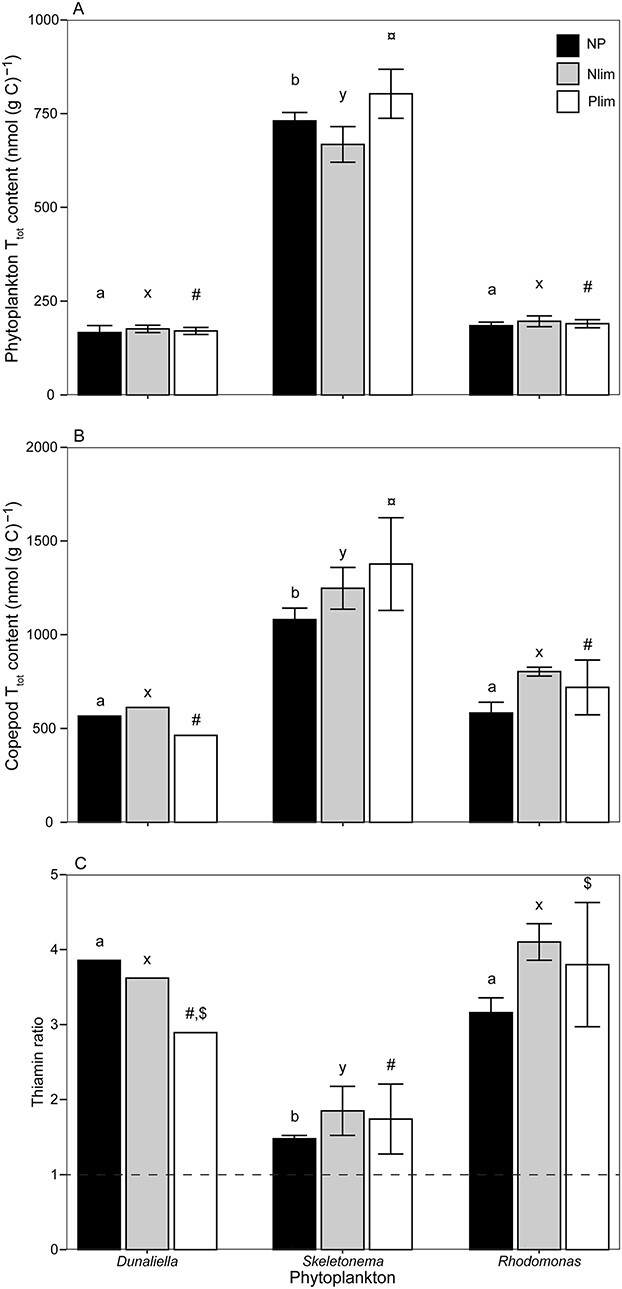
Total thiamin content in phytoplankton (**A**) and copepods (**B**), as well as thiamin ratio (**C**) for the different phytoplankton prey and nutrient treatments; NP (black), Nlim (gray) and Plim (white). Different letters indicate significant differences for different nutrient treatments, among phytoplankton prey, NP (a, b, c); Nlim (x, y, z); Plim (#, ¤, $). Dashed line show thiamin ratio of 1.

Thiamin content was highest in *Skeletonema* in all nutrient treatments compared to both *Dunaliella* (NP: *t* = 26.1, *p* < 0.001; Nlim: *t* = 20.9, *p* < 0.001; Plim: *t* = 27.2, *p* < 0.001) and *Rhodomonas* (NP: *t* = 24.2, *p* < 0.001; Nlim: *t* = 21.52, *p* < 0.001; Plim: *t* = 25.33, *p* < 0.001), Fig. 1A. Additionally, thiamin content did not differ significantly between *Dunaliella* and *Rhodomonas* when comparing different nutrient treatments (NP: *t* = 1.89, *p* = 0.63; Nlim: *t* = 1.70, *p* = 0.74; Plim: *t* = 1.88, *p* = 0.63). *Dunaliella* had similar thiamin content in all nutrient treatments (*F*_(2,5)_ = 0.32, *p* = 0.74) and thiamin content in *Rhodomonas* did not differ between treatments (*F*_(2,6)_ = 0.77, *p* = 0.50), Fig. 1A. For *Skeletonema*, there was a difference between nutrient treatments (*F*_(2,6)_ = 5.92, *p* = 0.04), with thiamin content being higher in the Plim treatment compared to the Nlim treatment (*t* = 3.44, *p* = 0.03), whilst thiamin content did not differ significantly between the other treatments, (Fig. 1A). This implies that thiamin prototrophy/auxotrophy did not have a large effect on the thiamin content for the investigated phytoplankton species, as the levels were at similar levels for *Rhodomonas* (auxotroph) and *Dunaliella* (prototroph).

Copepods fed with *Skeletonema* had higher levels of thiamin, irrespective of nutrient treatment, than copepods fed on *Dunaliella* (NP: *t* = 4.47, *p* < 0.05; Nlim: *t* = 4.64, *p* < 0.05; Plim: *t* = 7.48, *p* < 0.001) and on *Rhodomonas* (NP: *t* = 6.07, *p* < 0.01; Nlim: *t* = 3.85, *p* < 0.05; Plim: *t* = 6.40, *p* < 0.01), Fig. 1B. Copepods had similar thiamin content when they were fed with *Dunaliella* and *Rhodomonas*, in all nutrient treatments (NP: *t* = 0.18, *p* = 1; Nlim: *t* = 1.88, *p* = 0.63; Plim: *t* = 2.95, *p* = 0.17). Copepods fed on *Dunaliella* grown under different nutrient treatments did not show a difference in thiamin content (NP-Nlim: *t* = 0.44, *p* = 0.99; Nlim-Plim: *t* = 1.58, *p* = 0.79; NP-Plim: *t* = 1.14, *p* = 0.95), Fig. 1B. Thiamin content differed marginally for copepods fed on *Rhodomonas* in the different nutrient treatment (*F*_(2,6)_ = 4.96, *p* = 0.053), and was significantly different between NP and Nlim treatments (*t* = 3.12, *p* < 0.05), but not among the other nutrient treatments. Copepods fed *Skeletonema* had similar thiamin content irrespective of the nutrient treatments (*F*_(2,6)_ = 2.75, *p* = 0.16), Fig. 1B.

Thiamin ratio, which is the relative content of thiamin in the consumer and prey, was lowest in *Skeletonema* in NP and Nlim treatments compared to *Dunaliella* (NP: *t* = −5.35, *p* < 0.01; Nlim: *t* = −3.58, *p* < 0.05; Plim: *t* = −2.96, *p* = 0.16) and in all nutrient treatments when compared to *Rhodomonas* (NP: *t* = −5.98, *p* < 0.01; Nlim: *t* = −5.67, *p* < 0.01; Plim: *t* = −6.22, *p* < 0.01), Fig. 1C. Additionally, thiamin ratio was similar among *Dunaliella* and *Rhodomonas* irrespective of nutrient treatment (NP: *t* = 1.12, *p* = 0.96; Nlim: *t* = 0.69, *p* = 0.99; Plim: *t* = 4.09, *p* < 0.01). Due to problems in the analysis phase, thiamin ratio for *Dunaliella* grown under different nutrient condition could not be statistically investigated. Thiamin ratio for *Rhodomonas* from different nutrient treatments was similar (*F*_(2,6)_ = 3.08, *p* = 0.12), as well as for *Skeletonema* (*F*_(2,5)_ = 0.95, *p* = 0.45), Fig. 1C.

### Vitamer distribution in phytoplankton and copepods

**Fig. 2:**
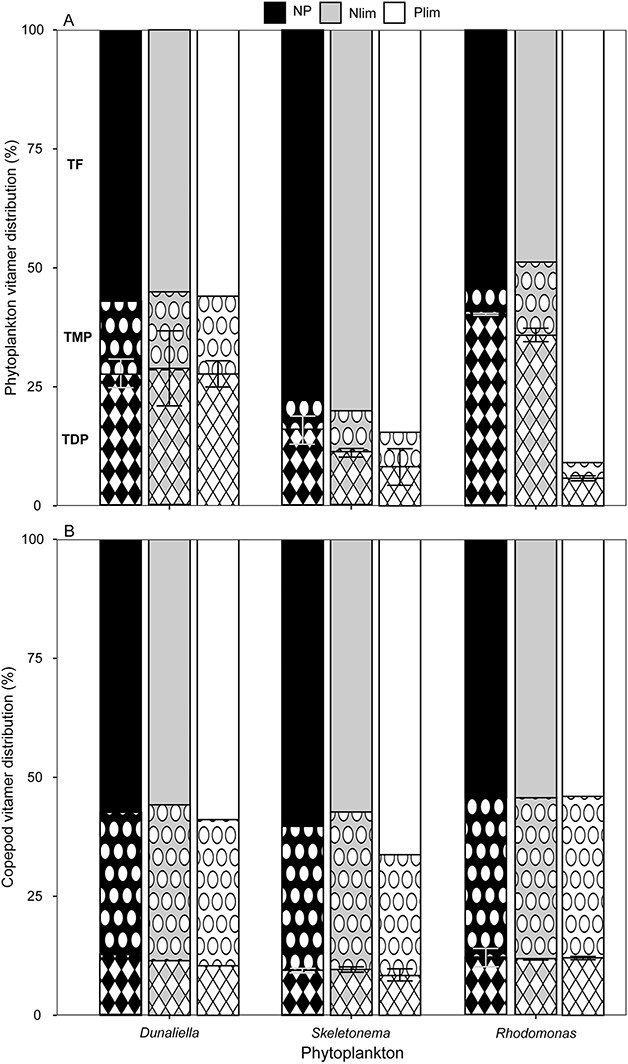
Vitamer distribution in in phytoplankton (**A**) and copepods (**B**), for the different phytoplankton prey and nutrient treatments; NP (black), Nlim (gray) and Plim (white). Different patterns show thiamin vitamer, TF (top, solid), TMP (middle, dots) and TDP (bottom, diamonds), e.g. left-most column in (A). Error bars show TDP ratio, with standard deviation.

The vitamer distribution in phytoplankton grown under different nutrient conditions varied, Fig. 2A, especially in terms of proportion of TDP (*F*_(8,17)_ = 40.85, *p* < 0.001). TDP ratio was found to be lowest in *Skeletonema* in all nutrient treatments compared to *Dunaliella* (NP: *t* = −3.62, *p* < 0.05; Nlim: *t* = −5.41, *p* < 0.01; Plim: *t* = −8.33, *p* < 0.001) and for the NP and Nlim treatment when comparing to *Rhodomonas* (NP: *t* = −6.04, *p* < 0.001; Nlim: *t* = −7.59, *p* < 0.001). For the Plim treatment, TDP ratio was similar among *Skeletonema* and *Rhodomonas* (*t* = 1.81, *p* = 0.67). Furthermore, TDP ratio was similar among *Dunaliella* and *Rhodomonas* in the NP and Nlim treatments (NP: *t* = 2.42, *p* = 0.33; Nlim: *t* = 1.39, *p* = 0.89) but lower for *Rhodomonas* in the Plim treatment (*t* = −10.14, *p* < 0.001), Fig. 2A. TDP ratio did not differ significantly among nutrient treatments for *Dunaliella* (*F*_(2,5)_ = 0.02, *p* = 0.98), whilst for *Rhodomonas* (*F*_(2,6)_ = 1 083, *p* < 0.001), thiamin ratio was lower in the Plim treatment compared to both NP (*t* = −41.43, *p* < 0.001) and Nlim (*t* = −39.07, *p* < 0.001). Also for *Skeletonema*, TDP ratio differed significantly between nutrient conditions (*F*_(2,6)_ = 5.07, *p* < 0.05), but the only significant difference was between NP-Plim (*t* = 3.18, *p* < 0.05) with the TDP ratio being lower in Plim (NP-Nlim: *t* = 1.53, *p* = 0.34; Plim-Nlim: *t* = −1.65, *p* = 0.30), Fig. 2A.

In copepods fed different phytoplankton species grown under different nutrient conditions, the vitamer distribution was very similar. The only significant difference was between *Skeletonema* and *Rhodomonas* in the Plim treatment, where the TDP ratio was lower in *Skeletonema*. For any other comparisons, among and within phytoplankton and nutrient conditions, the TDP ratio was similar in the copepods, Fig. 2B.

### Macro elemental composition in phytoplankton and copepods

*Dunaliella* had similar carbon content in all nutrient treatments (*F*_(2,6)_ = 0.47, *p* = 0.65), as was the carbon content in *Skeletonema* (*F*_(2,6)_ = 0.95, *p* = 0.44), and *Rhodomonas* (*F*_(2,6)_ = 1.86, *p* = 0.24), Table I. Carbon content was higher in *Rhodomonas* compared to *Skeletonema* in all nutrient treatments (NP: *t* = 5.74, *p* < 0.001; Nlim: *t* = 8.76, *p* < 0.001; Plim: *t* = 6.25, *p* < 0.001). When comparing *Rhodomonas* and *Dunaliella*, carbon content only differed significantly in the Nlim treatment, being higher for *Rhodomonas* (NP: *t* = 1.80, *p* = 0.68; Nlim: *t* = 4.03, *p* < 0.05; Plim: *t* = 2.52, *p* = 0.28), Table I. Carbon content was significantly lower in *Skeletonema* compared to *Dunaliella* in all nutrient treatments (NP: *t* = 5.74, *p* < 0.05; Nlim: *t* = 4.75, *p* < 0.01; Plim: *t* = 3.72, *p* < 0.05).

**Table I:** Macro elemental composition in phytoplankton. Different letters indicate significant differences for different nutrient treatments, among phytoplankton, NP (*a*, *b*, *c*); Nlim (*x*, *y*, *z*); Plim (#, *¤, $*)

Phytoplankton	Treatment	Abundance (10^6^ cells L^−1^)	POC (pg cell^−1^)	PON (pg cell^−1^)	POP (pg cell^−1^)
*Dunaliella*	NP	0.5 (±0.1)	49.5 (±7.1)*a*	8.9 (±1.2)*a*	0.6 (±0.1)*a*
	Nlim	0.5 (±0.1)	44.0 (±2.2)*x*	4.8 (±0.2)*x*	0.5 (±0.0)*x*
	Plim	0.5 (±0.1)	45.8 (±9.8)*#*	8.0 (±1.8)*#*	0.1 (±0.0)*#*
*Skeletonema*	NP	2.3 (±0.5)	29.2 (±4.2)*b*	4.4 (±0.8)*b*	0.2 (±0)*b*
	Nlim	2.3 (±0.2)	24.1 (±7.7)*y*	2.6 (±0.6)*y*	0.2 (±0)*y*
	Plim	2.0 (±0.1)	27.4 (±1.8)*¤*	3.8 (±0.3)*¤*	0.1 (±0)*¤*
*Rhodomonas*	NP	1.2 (±0.1)	62.6 (±2.1)*a*	10.3 (±0.2)*a*	0.2 (±0.1)*b*
	Nlim	0.7 (±0.2)	76.7 (±16.0)*z*	7.3 (±1.8)*x*	0.3 (±0.1)*y*
	Plim	1.3 (±0.1)	63.4 (±3.1)*#*	10.2 (±0.7)*#*	0.1 (±0.1)*#*,*¤*
Phytoplankton	Treatment		C:N (molar)	C:P (molar)	N:P (molar)
*Dunaliella*	NP		6.5 (±0.1)*a*	202.3 (±9.0)*a*	31.2 (±1.0)*a*
	Nlim		10.7 (±0.1)*x*	209.0 (±7.6)*x*	19.5 (±0.5)*x*
	Plim		6.7 (±0)*#*	813.3 (±48.5)#	121.1 (±6.5)#
*Skeletonema*	NP		7.7 (±0.2)*b*	471.1 (±43.4)*b*	61.1 (±4.1)*b*
	Nlim		10.6 (±0.8)*x*	395.0 (±0.4)*y*	37.1 (±4.7)*y*
	Plim		8.4 (±1.0)*¤*	874.7 (±133.3)#	103.3 (±4.1)#
*Rhodomonas*	NP		7.1 (±0.4)*a,b*	713.0 (±54.4)*c*	100.6 (±4.2)*c*
	Nlim		12.3 (±0.7)*x*	821.7 (±157.1)*z*	66.6 (±9.9)*z*
	Plim		7.2 (±0.4)*#*,*¤*	1572.0 (±47.6)*¤*	217.2 (±8.5)*¤*

Nitrogen content in *Dunaliella* differed significantly among treatments (*F*_(2,6)_ = 14.6, *p* < 0.01) and was lower in the Nlim treatment compared to both the NP and the Plim treatment (NP: *t* = 5.06, *p* < 0.01; Plim: *t* = 4.14, *p* < 0.05). Nitrogen content was similar between the NP and Plim treatment for *Dunaliella* (*t* = 0.92, *p* = 0.65), Table I. The trend was similar for *Rhodomonas* (*F*_(2,6)_ = 5.54, *p* < 0.05), with marginally significantly lower nitrogen content in the Nlim treatment compared to both NP (*t* = 2.93, *p* = 0.06) and Plim treatment (*t* = 2.84, *p* = 0.06), and with no significant differences between the NP and Plim treatment (*t* = 0.08, *p* = 1). Nitrogen content in *Skeletonema* differed among treatments (*F*_(2,6)_ = 7.55, *p* < 0.05) and was lower in the Nlim treatment compared to the NP treatment (*t* = 3.75, *p* < 0.05) and marginally significant for the Plim treatment (*t* = 2.75, *p* = 0.07). There was no significant difference in nitrogen content the NP and Plim treatment for *Skeletonema* (*t* = 0.99, *p* = 0.61). *Skeletonema* had significantly lower nitrogen content in all nutrient treatments compared to both *Dunaliella* (NP: *t* = 5.40, *p* < 0.01; Nlim: *t* = 4.83, *p* < 0.01; Plim: *t* = 5.64, *p* < 0.001) and *Rhodomonas* (NP: *t* = 6.59, *p* < 0.001; Nlim: *t* = 7.93, *p* < 0.001; Plim: *t* = 7.61, *p* < 0.001). Nitrogen content was similar between *Dunaliella* and *Rhodomonas* in all nutrient treatments (NP: *t* = 1.19, *p* = 0.95; Nlim: *t* = 3.1, *p* = 0.11; Plim: *t* = 1.96, *p* = 0.58), Table I.

Phosphorus content in *Dunaliella* differed significantly among treatments (*F*_*(*2,6)_ = 66.8, *p* < 0.001), and was lower in the Plim treatment compared to both the NP and the Nlim treatment (NP: t = 10.48, *p* < 0.001; Nlim: *t* = 9.46, *p* < 0.001) but similar between NP and Nlim treatment (*t* = 1.03, *p* = 0.59), Table I. Same pattern was true for *Rhodomonas* (F_(2,6)_ = 11.85, *p* < 0.01), with lower phosphorus content in the Plim treatment compared to both the NP (*t* = 4.06, *p* < 0.05) and the Nlim treatment (*t* = 4.36, *p* < 0.05) and at similar levels for NP and Nlim treatment (*t* = 0.30, *p* = 0.95). Also for *Skeletonema* the phosphorous content was significantly different among treatments (F_(2,6)_ = 14.3, *p* < 0.01), with lower levels in the Plim treatment compared to NP (*t* = 4.75, *p* < 0.01) and Nlim treatment (*t* = 4.51, *p* < 0.01) but similar when comparing NP and Nlim treatment (*t* = 0.25, *p* = 0.97), Table I. *Dunaliella* had significantly higher phosphorous content in all nutrient treatments compared to *Skeletonema* (NP: *t* = 8.59, *p* < 0.001; Nlim: *t* = 7.90, *p* < 0.001; Plim: *t* = 3.54, *p* < 0.05). *Rhodomonas* had lower phosphorous content than *Dunaliella* in the NP (*t* = 6.37, *p* < 0.001) and Nlim treatment (*t* = 5.11, *p* < 0.01) but for the Plim treatment the phosphorous content was similar (*t* = 2.02, *p* = 0.55). Phosphorous content was similar for *Skeletonema* and *Rhodomonas* for all nutrient treatments (NP: *t* = 2.22, *p* = 0.43; Nlim: *t* = 2.79, *p* = 0.19; Plim: *t* = 1.53, *p* = 0.83), Table I.

C:N ratio was higher in the Nlim treatment for all phytoplankton species, but the C:N ratio among phytoplankton within the Nlim treatment did not differ significantly. When comparing nutrient treatments, *Skeletonema* had a higher C:N ratio compared to *Dunaliella* in the NP (*t* = 3.69, *p* < 0.05) and Plim treatment (*t* = 5.00, *p* < 0.01), Table I. C:P ratio was higher in the Plim treatment for all phytoplankton, and within the Plim treatment C:P ratio was higher in *Rhodomonas* compared to *Dunaliella* (*t* = 7.01, *p* < 0.001) and *Skeletonema* (*t* = 6.30, *p* < 0.001), Table I. The pattern for C:P ratio was similar in all nutrient treatments; lowest in *Dunaliella*, higher in *Skeletonema* and highest in *Rhodomonas*. One exception was in the Plim treatment, where C:P ratio was similar in *Dunaliella* and *Skeletonema* (*t* = 0.70, *p* = 0.99), Table I. N:P ratio was highest in the Plim treatment for all phytoplankton, lowest in the Nlim treatment and intermediate in the N:P treatment, Table I. Like the C:P ratio, the N:P ratio was lowest in *Dunaliella*, higher in *Skeletonema* and highest in *Rhodomonas* in all nutrient treatments, Table I.

Copepods displayed similar carbon content, related to phytoplankton prey, at levels of between 0.7 and 1.8 μgC ind^−1^, Table II. Copepods fed *Skeletonema* tended to have lower carbon content than copepods fed *Dunaliella* and *Rhodomonas*, irrespective of nutrient treatment. Nitrogen content of copepods showed lower variability, where only copepods fed *Rhodomonas* in the NP treatment had slightly higher nitrogen content compared to copepods fed other phytoplankton prey in various nutrient treatments, Table II. C:N ratio displayed different patterns related to phytoplankton prey, with *Dunaliella* having similar ratios for all nutrient treatments. For *Skeletonema*, C:N ratio was similar for NP and Nlim treatment but lower in the Plim treatment, whilst for *Rhodomonas* the C:N ratio was lowest for NP, intermediate for Plim and highest for Nlim treatment, Table II.

**Table II:** Macroelemental composition in copepods fed different phytoplankton prey

Phytoplankton prey	Treatment	POC (μg ind^−1^)	PON (μg ind^−1^)	C:N (molar)
*Dunaliella*	NP	1.7	0.4	4.6
	Nlim	1.6	0.4	4.1
	Plim	1.8	0.4	4.9
*Skeletonema*	NP	0.9	0.2	4.9
	Nlim	0.8	0.2	4.8
	Plim	0.7	0.4	2.4
*Rhodomonas*	NP	1.5	1.3	1.3
	Nlim	1.1	0.2	5.3
	Plim	1.4	0.5	3.7

## DISCUSSION

Thiamin content of phytoplankton has been shown to be affected by salinity, temperature and light levels (Sylvander *et al.*, 2013). Also, additions of dissolved thiamin to phytoplankton cultures affect phytoplankton thiamin content (Fridolfsson *et al.*, 2020). Furthermore, copepod presence and the associated selective feeding, was found to affect thiamin content of a phytoplankton community (Fridolfsson *et al.*, 2018). It was hypothesized that nutrient limitation would influence the thiamin content of phytoplankton and zooplankton, based on results from multivariate and modeling studies (Ejsmond *et al.*, 2019; Majaneva *et al.*, 2020). In the present study, altered macronutrient ratios only caused diverse thiamin content in one of the investigated phytoplankton (*Skeletonema*) indicating that the effect of macronutrients on thiamin content in phytoplankton might be limited, in contrast to the expected outcome of a skewed nutrient ratio. Furthermore, as thiamin levels among the phytoplankton did not differ to any large extent, ability to produce thiamin *de novo* (thiamin prototrophy) of the phytoplankton did not appear to be any evident benefit for thiamin levels. Alternatively, it could be related to the requirements of the phytoplankton, as Droop (1958) found that *Skeletonema* was quite independent of thiamin supplement under favorable conditions and even when thiamin was needed it could be replaced by inorganic sulphide (Droop, 1958). Furthermore, thiamin precursors (thiazole and pyrimidine) have been shown to be a more bioavailable compound than the complete thiamin molecule, which also could have an effect on the thiamin content of the investigated phytoplankton (Droop, 1958; Gutowska *et al.*, 2017; Paerl *et al.*, 2018a). Most thiamin auxotrophs do not lack the complete thiamin synthesis pathway, but only one or more crucial thiamin precursors or degradation products of the synthetic pathway, termed Thiamin Related Compounds (TRCs) (Combs, 2012; Gutowska *et al.*, 2017; Kraft and Angert, 2017; Paerl *et al.*, 2018a; Suffridge *et al.*, 2020). In the bacterial community, recent findings show that most bacterioplankton in nature are reliant on one or more TRC and display a large seasonal variation (Gómez-Consarnau *et al.*, 2018; Paerl *et al.*, 2018b). Furthermore, a study using co-cultures found that prokaryotes can exchange TRC’s and that thiamin levels determine to which degree they interact (Sathe *et al.*, 2022).

Thiamin content in copepods differed mostly due to different phytoplankton prey and not macronutrient ratios which could imply that factors other than elemental stoichiometry are important for transfer of thiamin. Ingestion, digestion, and assimilation of prey could all play a role in the transfer of thiamin (Fridolfsson *et al.*, 2020) as well as macronutrients (Mitra and Flynn, 2005). Predators are able to buffer stoichiometric imbalances more than primary producers (Moorthi *et al.*, 2017). The ability of zooplankton to buffer nutrient levels has been proposed for thiamin as well, as seasonal variation in seston thiamin content was not associated with variation in zooplankton thiamin content (Fridolfsson *et al.*, 2019). Also, different thiamin content in various phytoplankton species did not render large differences in copepod thiamin content during feeding experiments (Fridolfsson *et al.*, 2020).

Whilst the effect of varying macronutrient ratios on total thiamin content was limited, the vitamer distribution differed among some nutrient treatments. The proportion of the active vitamer TDP was lower in the Plim treatment for *Rhodomonas* and *Skeletonema*, indicating that composition of macronutrients could affect the vitamer distribution in phytoplankton. This could indirectly be due to differences in growth rate among the nutrient treatments as proposed by Sylvander *et al.* (2013). Alternatively, there is a direct effect of limiting P levels in vitamer distribution in phytoplankton, but this requires additional studies. However, this pattern did not transfer into copepods, as the vitamer distribution was similar for copepods fed phytoplankton from various nutrient regimes. Thiamin must be converted to its unphosphorylated form (TF) to be taken up and enter the cells (Manzetti *et al.*, 2014), which could help explain why the vitamer distribution in prey is not mirrored in the consumer.

Even if the thiamin ratio differed among phytoplankton prey, it was > 1 for all phytoplankton and nutrient treatments. This could point towards an ability of copepods to accumulate thiamin for some periods and potentially serve as a thiamin-enriched food item, as long as the prey is edible/digestible. However, large filamentous cyanobacteria have been shown to contain high levels of thiamin (Sylvander *et al.*, 2013; Fridolfsson *et al.*, 2018, 2020), but the transfer to consumers was limited (Fridolfsson *et al.*, 2020).

Altered macronutrient ratios in the growth medium caused differences in both nutrient content (PON and POP cell^−1^) and nutrient ratios (C:N, C:P and N:P) in all phytoplankton species, demonstrating a plasticity related to supply of macronutrients. There were also differences among phytoplankton within nutrient treatments, illustrating that the macronutrient content of phytoplankton is species specific (Rhee and Gotham, 1980). C:N ratio was lower in copepods compared to its phytoplankton prey in all nutrient treatments as well as less variable, conforming to the theory that consumers can buffer stoichiometric imbalances (Sterner and Elser, 2002; Mitra and Flynn, 2005; Hessen *et al.*, 2013).

It has been proposed that phytoplankton fatty acid profiles, especially highly unsaturated fatty acids (HUFA’s) are important when assessing food web transfer (Brett and Müller-Navarra, 1997), in addition to levels of macronutrients (C, N and P) as well as cell morphology, and being ingestible and digestible. We suggest that micronutrients (e.g. thiamin) also offer additional insights into food web efficiency as it is essential for all living organisms and have been shown to shape several ecological processes (Kraft and Angert, 2017).

## CONCLUSIONS

Thiamin originates from the lower trophic levels in the aquatic food web, e.g. bacteria, phytoplankton and fungi. In this realm, both thiamin prototrophs and auxotrophs co-exist. Our study provides new insights into thiamin-transfer from phytoplankton to copepods, under various nutrient replete and limiting conditions. Thiamin content of phytoplankton was not vastly affected by various macro-nutrient conditions, nor was the associated thiamin content in copepods. However, thiamin content differed among phytoplankton as well as among copepods fed different prey, being highest in *Skeletonema* treatments. Macro-elemental content of phytoplankton and copepods were affected by alternate nutrient regimes, indicated by both content and nutrient ratios. Moreover, nutrient content of phytoplankton was species-specific as *Skeletonema* displayed lower C, N and P content in all nutrient treatments. Consequently, *Skeletonema* was found to have the highest thiamin content but also the lowest macronutrient contents and enabled a higher thiamin content on copepods feeding on *Skeletonema*. However, the thiamin ratio was lowest in for *Skeletonema*, meaning that while seeming to be the most valuable thiamin resource when assessing content, the transfer appeared to be sub-optimal. Potentially this is related to factors other than macronutrient and thiamin content, e.g. ingestion, digestion or fatty acid profiles. Overall, this suggests that phytoplankton species composition and associated characteristics would be more important for the thiamin transfer than macronutrient conditions.

## Data Availability

Data will be available upon request.
